# A diminutive perivascular epithelioid cell tumor in the colon

**DOI:** 10.1002/deo2.390

**Published:** 2024-05-20

**Authors:** Naoki Sugimura, Daizen Hirata, Mineo Iwatate, Santa Hattori, Mikio Fujita, Wataru Sano, Takahiro Fujimori, Yasushi Sano

**Affiliations:** ^1^ Gastrointestinal Center & Institute of Minimally‐Invasive Endoscopic Care (i‐MEC) Sano Hospital Hyogo Japan; ^2^ Pathology Division Sano Hospital Hyogo Japan

**Keywords:** angiomyolipoma, colon, diminutive, mesenchymal tumor, PEComa

## Abstract

Perivascular epithelioid cell tumor (PEComa) is a rare mesenchymal tumor. Some papers have reported that colonoscopy could be used to treat PEComa with a predominantly pedunculated polyp, whereas surgical intervention is often required for cases with submucosal‐type tumors. These findings suggest that the morphology of PEComa changes dramatically with disease progression. Because of the rapid progression of PEComa, endoscopic treatment remains challenging, and early‐stage PEComa morphology is not well understood. A 64‐year‐old man presented to our hospital for a follow‐up colonoscopy after undergoing multiple polypectomies. He had a medical history of colorectal adenoma and prostate cancer. A 4‐mm pale blue elevated but not pedunculated lesion was observed in the transverse colon, an area where he had not had polyps previously. Since no epithelial change was observed, the presence of a submucosal tumor, such as a gastrointestinal stromal tumor, was suspected. Cold snare polypectomy was performed, and the lesion was completely resected. Histological evaluation using hematoxylin and eosin staining identified that the submucosal tumor included thickened vascular walls and adipose tissue. Although fragmented due to significant degeneration, spindle‐shaped cells staining positive for smooth muscle actin were observed within and surrounding the unstructured hyalinized tissue with calcifications. Based on these findings, the lesion was diagnosed as angiomyolipoma, a subtype of PEComa. Complete resection was confirmed by histopathology. To our knowledge, this PEComa is the smallest of any PEComa reported in the literature. Our finding provides valuable insights into the very early stage of colorectal PEComas.

## INTRODUCTION

Perivascular epithelioid cell tumor (PEComa) is a rare mesenchymal tumor that occurs mostly in females, and it can manifest in various anatomical locations including the uterus, skin, retroperitoneum, and colon. In 2002, PEComa was defined by the World Health Organization (WHO) as a mesenchymal tumor composed of histologically and immunohistochemically distinctive perivascular epithelioid cells (PECs).^1^ As PEComa tumors originate from PEC‐derived mesenchymal cells, they are included in a variety of tumors such as angiomyolipoma, clear cell sugar tumor of the lung, lymphangioleiomyomatosis, clear cell myomelanocytic tumor of the falciform ligament/ligamentum teres, abdominopelvic sarcoma of perivascular epithelial cells, and primary extrapulmonary sugar tumor.^2^ Doyle et al. reported 35 cases of PEComas in distinct sites of the gastrointestinal tract, including colon (54%), small intestine (35%), and stomach (6%). Notably, 13 patients (37%) developed metastases during follow‐up, implying the aggressive nature of this disease.^3^

To date, the clinical features of colon PEComa remain unclear due to its rarity. Some papers have reported that colonoscopy could be used to treat PEComa with a predominantly pedunculated polyp,^4^ whereas surgical intervention is often required for cases with submucosal‐type tumors.^5^ These findings suggested that the morphology of PEComa changes dramatically along disease progression. However, due to the rapid progression of PEComa, endoscopic treatment remains challenging with an inadequate understanding of its morphology at an early stage.

## CASE REPORT

**FIGURE 1:**
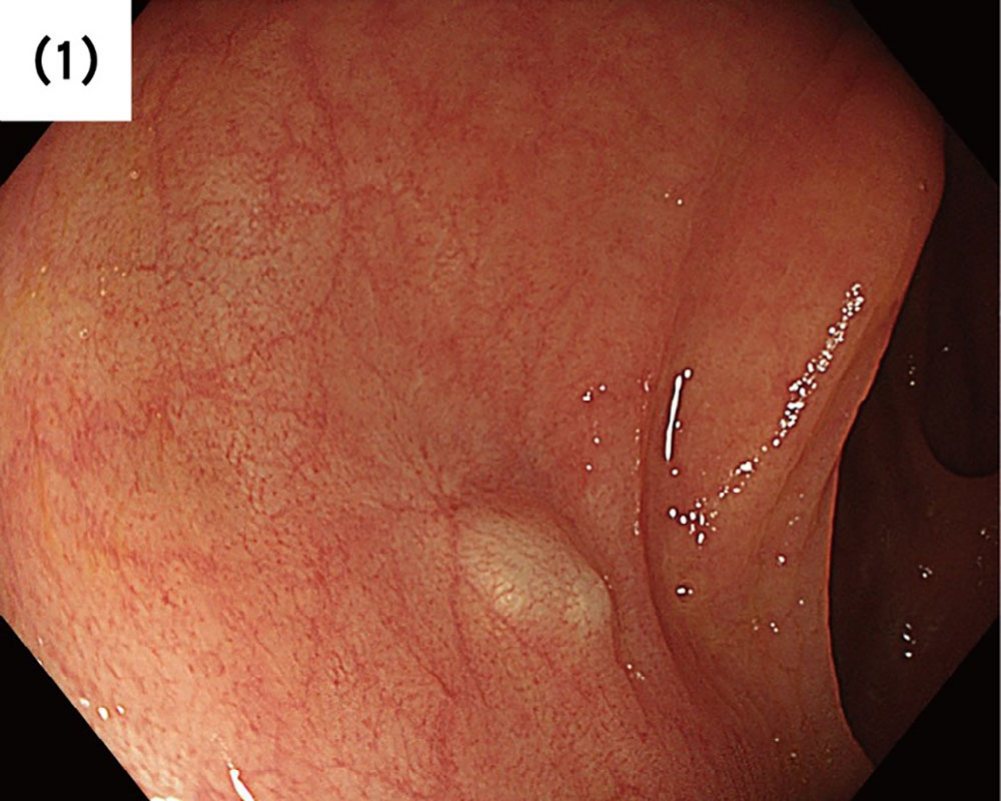
A 4‐mm pale blue elevated but not pedunculated lesion was observed in the transverse colon. No epithelial change was observed.

In this case, a 64‐year‐old man presented to our hospital for a follow‐up colonoscopy after undergoing multiple polypectomies. He had a medical history of colorectal adenoma and prostate cancer. A 4‐mm pale blue elevated but not pedunculated lesion was observed in the transverse colon, an area where no polyps were observed previously (Figure 1). Since no epithelial change was observed, the presence of a submucosal tumor, such as a gastrointestinal stromal tumor, was suspected. Cold snare polypectomy was performed, and the lesion was completely resected.

**FIGURE 2:**
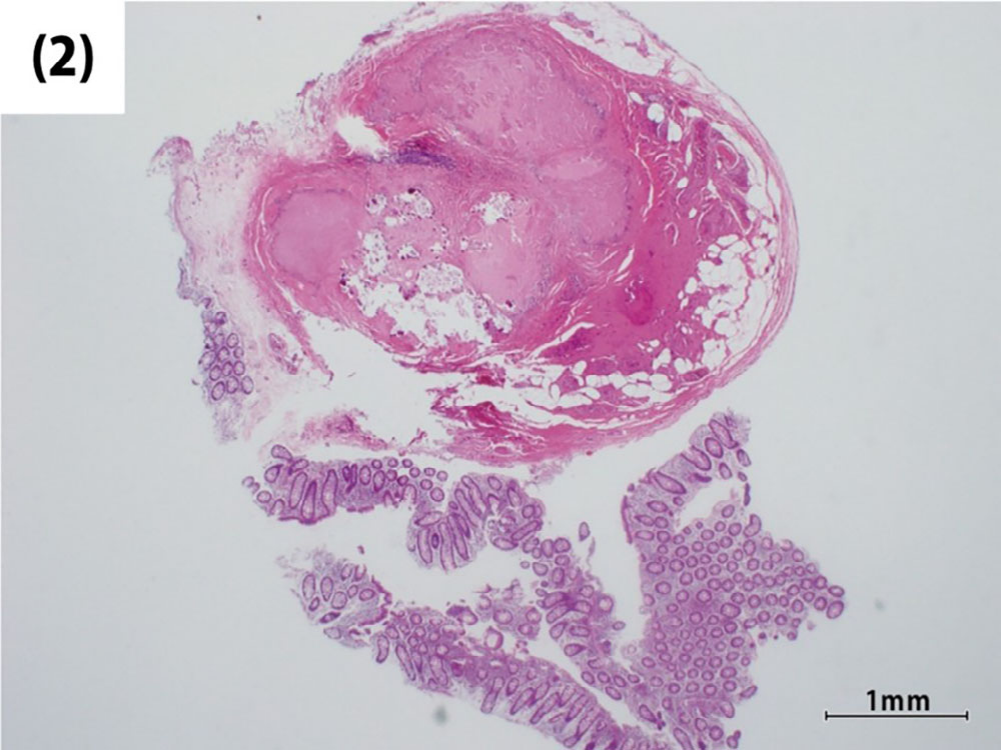
Histological image stained with hematoxylin and eosin revealed the presence of unstructured hyalinized tissue with calcifications within the submucosal tumor, which was observed in approximately 60% of the lesion.

**FIGURE 3:**
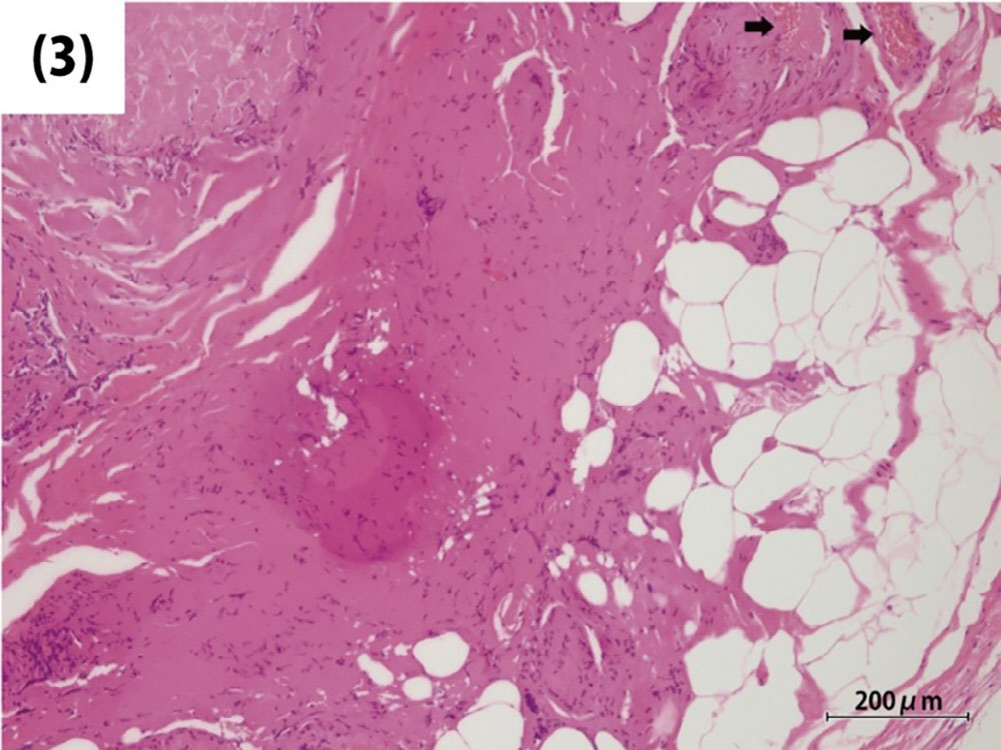
Thickened vascular walls (arrows) and adipose tissues observed in the lesion.

**FIGURE 4:**
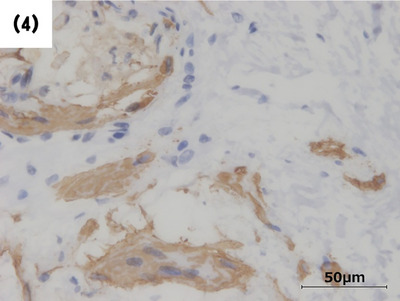
Although fragmented due to significant degeneration, spindle‐shaped smooth muscle actin‐positive cells were observed within and surrounding the unstructured hyalinized tissue.

Histological images stained with hematoxylin and eosin revealed that the submucosal tumor (Figure 2) included thickened vascular walls (arrows) and adipose tissue (Figure 3). In this case, the presence of unstructured hyalinized tissue with calcifications was observed in approximately 60% of the lesion (Figure 2), making it difficult to definitively identify smooth muscle composed of epithelioid cells. In histopathological diagnosis, WHO defines PEComa as a mesenchymal neoplasm with positive immunohistochemical staining for specific markers, including smooth muscle and melanocytic markers.^1^ Therefore, immunohistochemical staining was conducted for smooth muscle actin and melanocytic markers including HMB45 and Melan‐A. Although fragmented due to significant degeneration, spindle‐shaped smooth muscle actin‐positive cells were observed within and surrounding the unstructured hyalinized tissue (Figure 4). The melanocytic markers were negative. Based on the three components: blood vessels, mature fat cells, and smooth muscle cells, the lesion was thereby diagnosed as angiomyolipoma, a subtype of PEComa. Complete resection was confirmed by histopathology.

After the treatment of colorectal PEComa, additional investigations including upper gastrointestinal endoscopy, chest X‐ray examination, and abdominal computed tomography scan were conducted, but no lesions suggestive of malignancy or metastasis were identified, except for the previously known prostate cancer. Furthermore, the levels of serum tumor markers including cancer embryonic antigen, cancer antigen 19–9, as well as other hematological tests, were within normal limits.

## DISCUSSION

PEComa presents significant challenges for diagnosis and management due to its rarity, varied clinical behavior, and morphological characteristics. The gastrointestinal tract, particularly the colon, is the second most common location of PEComas, accounting for 20%–25% of all PEComa cases.^6^ In previous studies, PEComas with a size of 1–2 cm exhibited a pedunculated polypoid morphology.^4^ Here, a 4‐mm lesion was observed in the transverse colon with an elevated but not pedunculated morphology, which may be indicative of a pre‐pedunculated polypoid morphology. The lesion was completely resected by cold snare polypectomy. Histologically, the tumor consisted of three components: blood vessels, mature fat cells, and smooth muscle cells, leading to a diagnosis of angiomyolipoma, a subtype of PEComa. The great majority of angiomyolipomas originate in the kidney, while angiomyolipoma of the colon is extremely rare.^5^

Folpe et al. previously proposed a classification for PEComa malignancy based on the analysis of 26 cases in soft tissues and gynecological areas, categorizing them as “benign,” “of uncertain malignant potential,” and “malignant.”^7^ Under this classification, the likeliness of malignant behavior can be estimated by means of several characteristics including tumor size >5 cm, necrosis, infiltrative growth pattern, marked nuclear atypia, cellularity, and high mitotic activity >1/50 HPF, which are associated with increased risk of malignant behavior. In the current PEComa case, there were no observations suggestive of malignancy, thus it was classified as benign. The complete resection of the lesion in our case underscores the importance of early detection and endoscopic intervention in managing PEComa. Given the reported aggressive behavior of PEComa with metastatic potential, prompt diagnosis and treatment are essential to improve patient outcomes.

In conclusion, to our knowledge, we reported a case with the smallest PEComa among all literatures, regardless of the organ. This finding provides valuable insights into colorectal PEComas at a very early stage. Further research is necessary to evaluate the occurrence, morphological alterations accompanied by tumor growth, precise diagnosis, and indications for endoscopic therapy in managing this rare disease.

## CONFLICT OF INTEREST STATEMENT

None.
